# Iatrogenic intralenticular broken steroid implant: a case report

**DOI:** 10.1186/s13256-019-2064-1

**Published:** 2019-05-06

**Authors:** Jun Fai Yap, Yong Zheng Wai, Qi Xiong Ng, Lik Thai Lim

**Affiliations:** 1 Hospital Tuanku Ja’afar Seremban, Seremban, Negeri Sembilan Malaysia; 2 Universiti Malaysia Sarawak (UNIMAS), Kota Samarahan, Sarawak Malaysia

**Keywords:** Ozurdex™ implant, Steroid implant, Intralenticular, Uveitis, Case report

## Abstract

**Background:**

This is a case report of an iatrogenic intralenticular broken steroid (Ozurdex™) implant in a patient with uveitis. There are only a few case reports on broken Ozurdex™ implants in the vitreous cavity, with none of them involving the crystalline lens. A few authors have described the accidental injection of an Ozurdex™ implant into the crystalline lens, but all of the implants remained in one piece in the lens and none of them were broken. We report an unusual case of an Ozurdex™ implant which was injected inadvertently into the crystalline lens, resulting in a broken Ozurdex™ implant with an entry and exit wound through the posterior capsule of the lens.

**Case presentation:**

An ophthalmic trainee performed an Ozurdex™ intravitreal injection into a 48-year-old Asian man’s right eye under aseptic conditions. This patient was then followed up for further management. On day 7 post-procedure, a slit lamp examination revealed that the Ozurdex™ implant was injected into the intralenticular structure of his right eye and had fractured into two pieces. The posterior capsule of the right lens was breached, with one half of the Ozurdex™ implant stuck at the entry and the other stuck at the exit wound of the posterior capsule. This patient underwent right eye cataract extraction and repositioning of the fractured implant; he made an uneventful recovery.

**Conclusions:**

Ophthalmologists should be aware of the potential risk of injecting an Ozurdex™ implant into an anatomical structure other than the vitreous cavity. Adequate training and careful administration of the Ozurdex™ implant are necessary to avoid such a complication, which fortunately is rare.

## Background

Ozurdex™ (Allergan Pharmaceuticals) is a sustained-release biodegradable steroid ocular implant containing 0.7 mg dexamethasone. It is licensed in the UK and Malaysia for the treatment of macular edema secondary to retinal vein occlusion and non-infectious posterior segment uveitis [1]. The ZERO study [2] conducted in Germany was designed to evaluate the safety and reliability of intravitreal Ozurdex™ injections. The result of the study revealed that there were no broken Ozurdex™ implants and no intraoperative lens injuries were reported [2]. There are only a few case reports on broken Ozurdex™ implants in the vitreous cavity, with none of them involving the crystalline lens [3–5]. A few authors have described the accidental injection of an Ozurdex implant into the crystalline lens, but all of the implants remained as one piece in the lens and none of them were broken [6–9].

We report an unusual case of an Ozurdex™ implant which was injected unintentionally into the crystalline lens resulting in a broken Ozurdex™ implant with an entry and exit wound through the posterior capsule of the lens.

## Case presentation

A 48-year-old Asian man was treated with Ozurdex™ intravitreal injection for uveitis secondary to sarcoidosis in his right eye. The diagnosis of sarcoidosis was presumptively made as he had compatible clinical evidence of hypercalcemia with raised serum angiotensin-converting enzyme (ACE) and radiological manifestations, such as bilateral hilar adenopathy, on chest X-ray after excluding other diseases that may present similarly.

He initially complained of seeing floaters over his right eye. The visual acuity of his right eye was 6/24 whereas his left eye was normally recorded as 6/6. The initial intraocular pressure (IOP) for both eyes were within the normal range (16 mmHg bilaterally). A slit lamp examination revealed right eye perivascular sheathing and Standardization of Uveitis Nomenclature (SUN) grade 2 vitritis changes. Despite intensive topical steroid treatment for 6 months, the vitritis persisted with no significant IOP increment; hence, an Ozurdex™ intravitreal injection was planned.

An ophthalmic trainee performed the procedure under aseptic conditions using the standard technique under supervision. The trainee did not notice significant recoil force generated throughout the Ozurdex™ intravitreal injection.

**Fig. 1:**
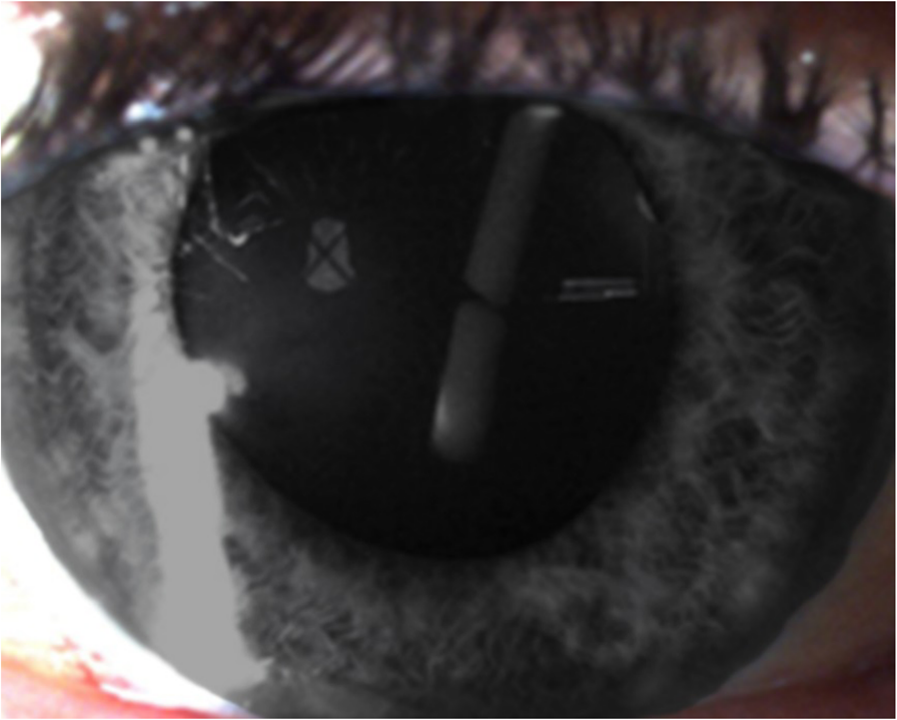
The intravitreal injection was done inferotemporally; hence, the direction of the broken Ozurdex™ implant

**Fig. 2:**
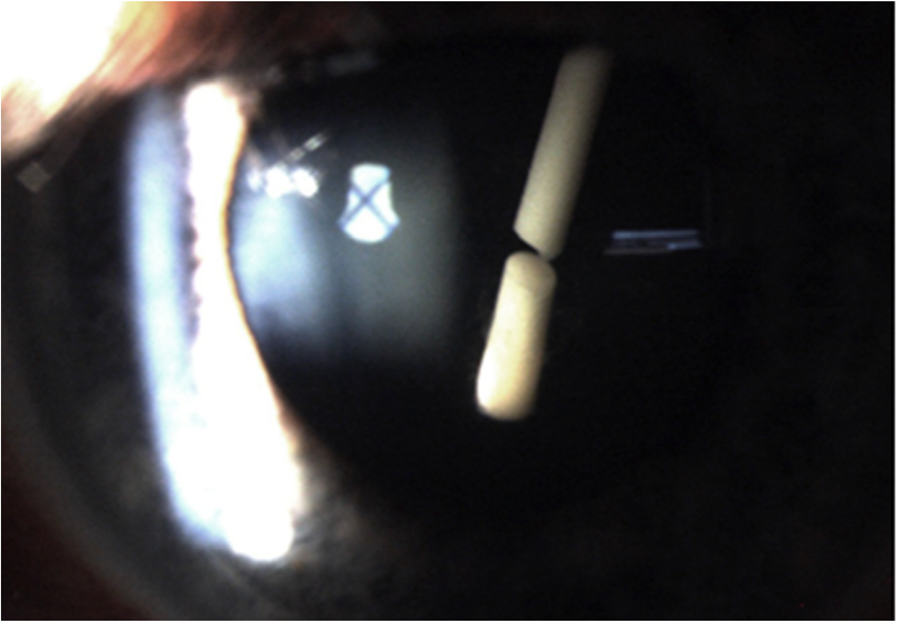
Although this might not be apparent in the picture, a cursory examination revealed an entry site represented by the posterior capsular breach and an exit site posterior capsular breach of the shorter and longer broken implants, respectively

**Fig. 3:**
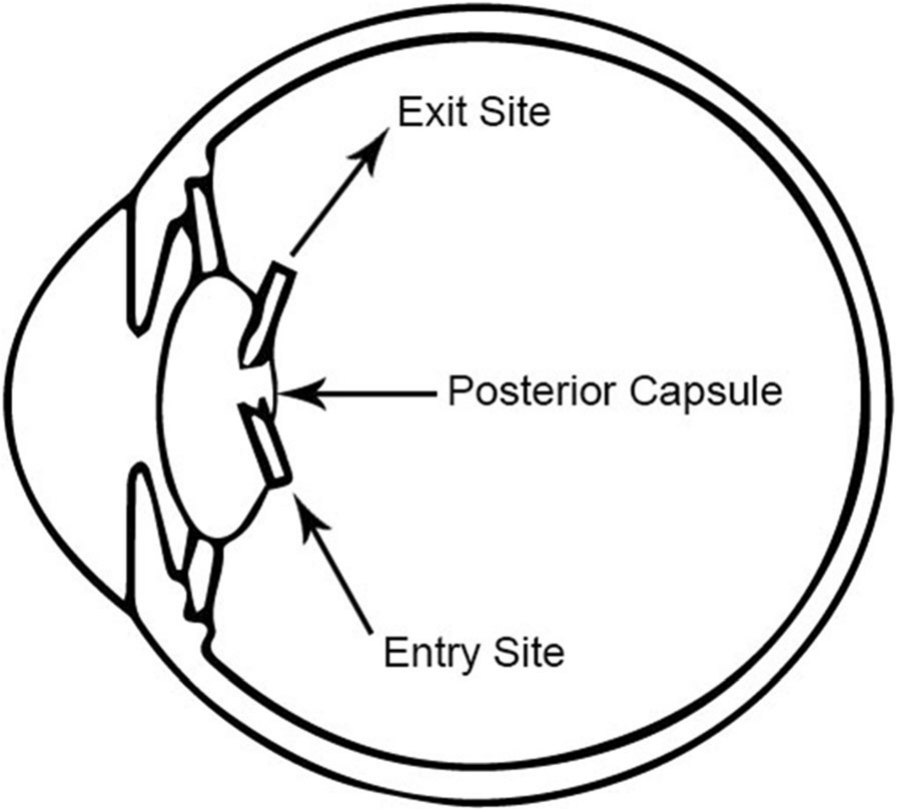
A schematic figure to show in greater detail the extent and position of the entry and exit points of the broken Ozurdex™ implant injected pars plana. The Ozurdex™ implant was broken during the insertion process when this unwanted intralenticular event occurred

On day 7 post-procedure, this patient had a routine follow-up visit at the medical retina clinic and the injected Ozurdex™ implant was found to be broken into two pieces and located intralenticularly with the entry site at the inferotemporal region, breaching the posterior capsule of the lens (Figs. 1 and 2). No damage to the surrounding eye structures or cataract formation was observed. A schematic diagram showing a sagittal view of the eyeball (Fig. 3) aids understanding of the entry and exit points of the broken implant into the lens. The visual acuity of our patient’s affected eye at that clinic review was 6/9 on Snellen chart.

The proposed mechanism of this intralenticular Ozurdex™ implant fracture with both entry and exit sites at different areas of the posterior capsule is the possible anterior rotation and recoil force generated as the Ozurdex™ implant was injected intravitreally with the direction of the injector pointed unintentionally toward the lens (instead of pointed toward the direction of the optic disc), thereby resulting in the above clinical findings. In order to reduce the recoil force, we suggest the Ozurdex™ implant intravitreal injection be done toward the direction of the optic disc.

The posterior capsular breach occurred in two sites of the posterior capsule (as shown in Fig. 2), encroaching on the visual axis; a decision was made to proceed with the right eye cataract extraction and to reposition the fractured implant. A dispersive ophthalmic viscoelastic device (OVD) was used to help protect the corneal endothelium during phacoemulsification. The right eye natural lens was successfully removed as per usual phacoemulsification steps using divide and conquer technique. The broken Ozurdex™ implant was repositioned through the already-breached posterior capsule into the vitreous cavity (which was the initial rightful anatomical position for the Ozurdex™ implant) after the natural lens removal without vitreous loss. An intraocular lens was successfully placed in the capsular bag, after the broken Ozurdex™ implant was repositioned into the vitreous cavity. Finally, a circular posterior capsule capsulorhexis was performed prior to closing up.

At 3-month follow-up, the lens remained in place and unaided visual acuity of our patient’s right eye was 6/9 with no macular edema. No posterior capsular opacification occurred and the IOP of both eyes were not raised. No endophthalmitis or retinal detachment signs were noted. The uveitis resolved completely at 3-month follow-up with no vitritis changes on fundus examination, with right eye best corrected visual acuity of 6/6 on Snellen chart.

## Discussion and conclusions

To the best of our knowledge, this is the first case of iatrogenic intralenticular broken steroid (Ozurdex™) implant with an entry and exit site at different areas of the posterior capsule.

Despite a previous case report stating observation of a stable intralenticular broken Ozurdex™ implant [6], a surgical approach was deemed appropriate due to the unusual nature of the intralenticular broken implant which was encroaching on the visual axis and the increased risk of cataract formation. With the emergence of microsurgical implant intravitreal injection procedures, clinicians should be aware of the potential risk of injecting the implant into an anatomical space other than the vitreous cavity. It is important to emphasize the fact that any intravitreal injection should be pointed toward the direction of the optic disc and not in the direction of the lens. In our case, the fracture of an Ozurdex™ implant did not affect its efficacy as the vitritis had resolved completely after the phacoemulsification. Adequate training and careful administration of the Ozurdex™ implant are necessary to avoid such a rare complication.
